# Five year cost savings of a multimodal treatment program for child sexual abuse (CSA): a social return on investment study

**DOI:** 10.1186/s12913-022-08267-w

**Published:** 2022-07-09

**Authors:** Hannah Pazderka, Matthew Reeson, Wanda Polzin, Jonathan Jin, Gary Hnatko, Yifeng Wei, Vincent I. O. Agyapong, Andrew J. Greenshaw, Arto Ohinmaa, Peter H. Silverstone

**Affiliations:** 1 Department of Psychiatry, University of Alberta, 1E7.17 Mackenzie Centre, 8114 -112 Street, Edmonton, Alberta T6G 2B7 Canada; 2 Be Brave Ranch, Centre for Treatment of Child Sexual Abuse, Edmonton, Canada; 3 CASA Child, Adolescent and Family Mental Health, Edmonton, Canada; 4 Department of Psychiatry, Dalhousie University, Halifax, Canada; 5 School of Public Health, University of Alberta, Edmonton, Canada

**Keywords:** Social return on investment, Child sexual abuse, Child and adolescent mental health, Mental health treatment, Public policy

## Abstract

**Background:**

Specialized mental health services for the treatment of Child Sexual Abuse (CSA) are generally expensive and labour intensive. They require a trauma-informed approach that may involve multiple services and therapeutic modalities, provided over the course of several months. That said, given the broad-ranging, long term negative sequelae of CSA, an evaluation of the cost-benefit analysis of treatment is clearly justified.

**Methods:**

We performed a Social Return on Investment (SROI) analysis of data gathered as part of the treatment program at the Be Brave Ranch in Edmonton, Canada to determine the value-for-money of the services provided. We endeavoured to take a conservative, medium-term (5 year) perspective; this is in contrast to short term (1–2 year) effects, which may rapidly dissipate, or long term (15–20 year) effects, which are likely diffuse and difficult to measure. As such, our analysis was based on an average annual intake of 100 children/adolescents (60:40 split) and their families, followed over a five-year timeframe. Financial proxies were assigned to benefits not easily monetized, and six potential domains of cost savings were identified.

**Results:**

Our analyses suggest that each dollar spent in treatment results in an average cost savings of $11.60 (sensitivity analysis suggests range of 9.20–12.80). The largest value-for-money was identified as the domain of crisis prevention, via the avoidance of rare but costly events associated with the long term impacts of CSA. Somewhat surprisingly, savings related to the area of criminal justice were minimal, compared to other social domains analysed. Implications are discussed.

**Conclusions:**

Our results support the cost effectiveness of the investment associated with specialized, evidence-based early interventions for CSA. These approaches alleviate severe, negative outcomes associated with CSA, resulting in both economic savings and social benefits. These findings rest upon a number of assumptions, and generalizability of these results is therefore limited to similar programs located in comparable areas. However, the SROI ratio achieved in this analysis, in excess of $11:1, supports the idea that, while costly, these services more than pay for themselves over time.

## Introduction

Childhood sexual abuse (CSA) is an adverse childhood experience (ACE) that represents an area of substantial, growing clinical impact and associated cost, and population studies of ACE scores suggest that approximately 11% of adults identify it as an adverse early event, which rises to 16% amongst people living in poverty [1]. Estimates suggest that roughly 1 in 12 men and 1 in 5 women have experienced at least one instance of CSA [2, 3], although local studies have suggested rates may be as high as 1 in 3 [4]. Understanding the true extent of the problem is challenging, as up to 95% of CSA incidents go unreported to official authorities [5].

Early life trauma can have devastating effects on brain development [6, 7]. It increases risk for subsequent mental health problems, with 30–40% of survivors experiencing major depressive disorder with suicidal thoughts [8], and nearly 20% experiencing panic disorder [9]. Individuals are also more than twice as likely to develop alcohol dependence [8]. These chronic issues are all associated with higher health costs throughout the lifespan. Moreover, these costs do not even begin to address chronic physical health conditions (including diabetes, cancer, and heart disease) which a recent meta-analysis of 37 separate studies has identified as related to high ACE scores [10]. These analyses provide broader context to the role that early trauma plays in health. CSA also results in both increased social costs (higher rates of divorce, prostitution, unwanted pregnancy, homelessness, criminal activity, and incarceration) and economic losses (lost productivity, lower educational achievement, poorer job prospects, and problems forming stable partnerships) [11]. These difficulties result in markedly lower quality of life, with increased reports of somatization and psychiatric symptoms that correlate with severity of abuse [12]. Accordingly, it is estimated that lifetime costs associated with CSA are between $75,000–280,000 USD (approximately 95,000–350,000 CAD for males and females respectively), and in cases ultimately resulting in suicide, these estimates jump to more than $1.1 million (almost 1.4 M CAD) [13]. Together, these calculations suggest that effective early intervention for CSA, successfully mitigating these negative impacts, will produce reduced lifetime cost expenditures.

### Program description

One evidence-based program for treatment of CSA is the Little Warriors *Be Brave Ranch* (BBR) in Alberta, Canada. Operating since fall 2014, the BBR provides a secure, remote facility where children and adolescents receive 200h hours of targeted, multi-modal, episodic treatment in a welcoming, camp-like setting (community-based therapies, by contrast, are generally limited to 1–2 hours per week). The program is unique in that it is funded entirely via donations and receives no provincial healthcare funding. This approach makes understanding value-for-money particularly critical for the success of this program.

Intended to be both wrap-around and holistic, the BBR approach includes trauma-focused cognitive behavioural therapy, dialectical behavioural therapy-informed approaches, psychoeducation, mindfulness, art therapy, music therapy, animal assisted therapy, and yoga. Small cohorts go through treatment stages together, building relationship skills and trust; finding other youngsters who have had similar experiences also helps the individual to feel less alone and reduces the stigma and shame associated with CSA. Thus, the program strives not only to relieve distressing symptoms, but addresses the underlying trauma, helping teach relational skills and promoting a sense of balance and perspective for the individual and their family. The approach has shown promising early outcomes, in terms of improved cognition [14] and symptom reduction (depression, anxiety, and PTSD) [15]. Beyond positive short-term effects, we posit that improvements in forming stable relationships, decision making, and emotional functioning have broader, long-term implications, resulting in reduced long-term societal costs. However, such programs are costly given relatively high expenses including both accommodations and human resources-intensive activities, and their value may be questioned from an economic perspective.

### Social Return on Investment methodology

We suggest opportunities for early trauma intervention can provide good value-for-money, relevant in this economic climate where many competing causes seek support. This investigation underpins a business case detailing potential cost savings for programs specializing in CSA. Yet these costs can be difficult to quantify, given the benefits under consideration are “social”, and therefore intangible. Thus, in order to estimate the economic cost savings of the treatment of CSA, a Social Return on Investment (SROI) was calculated. The SROI is an estimate of cost savings obtained from a certain program or service, presented as a ratio of the net present value of benefits to investment; it follows a structured, transparent approach that produces a cost estimate of program value [16, 17]. It does this using financial proxies to estimate the value of benefits not easily monetized [18]. Hence, SROI is appropriate for evaluating complex, far-reaching social issues such as CSA.

Somewhat surprisingly, no previous SROI studies of CSA treatment were located in our search of the literature nor were any identified in a 2020 systemic scoping review [19]. Overall, we found very few programs aimed at examining child or adolescent mental health more generally. One study looked at the potential benefits of involvement in a circus-arts training program [20], and identified improvement in four key areas of mental health: self-esteem, confidence, socialisation and stress-relief; this study estimated approximately $7 of social return on every dollar invested in the program. Finding benefits in a similar range, another study looked at the addition of a schoolwide music program meant to build social cohesion found a return of approximately £6.7:1 [21]. Another study examined the merits of a “cancer camp” for children and adolescents living with cancer (including survivors and siblings) [22]; this study found only a $4 social value return for every dollar invested - although it should be noted that their program did not appear to offer any type of therapy or focus on any specific social change. Conversely, in their SROI of Children’s Aid community schools in New York [23], in which two different sites were examined, the SROIs were quite a bit higher – between 10.3–14.8:1. More in line with what is provided at the BBR, those schools offered services in four key areas: onsite or school-linked health/mental health services; expanded learning opportunities (e.g. after-school and summer programs); parent education and engagement; family support services. However, none of these programs focused specifically on children with a history of sexual trauma. Accordingly, the goal of this study was to use a societal perspective to calculate potential cost savings realized by helping children and adolescents who have been the survivors of sexual abuse using an SROI methodology.

## Methods

At BBR, data is collected routinely as part of intake and ongoing monitoring of clinical programming. Data analysis for this study was approved by the University of Alberta Human Research Ethics Committee (Ethics review number: Pro00089614).

To evaluate a potential cost savings, one must determine the current budget. Exact annual costs per patient are difficult to calculate for a number of reasons. First, the BBR uses a staggered intake, meaning individuals enter treatment at different points in the year (cohorts); thus, in any given fiscal year there are some individuals who are just beginning treatment while others are in the midst of completing it. Moreover, the first round of treatment is the longest and most involved, thus it is also the most resource-intensive. Additionally, not all individuals who start treatment ultimately complete the program, again complicating the calculation. Finally, costs for some individuals can vary substantially, due to factors such as distance (the BBR accepts patients from across Canada and covers all associated travel costs), and these may likewise change over time. That said, on average BBR’s costs for year-long treatment range from $15,000 CDN for adolescents, to $20,000 CDN for children (variance reflecting program differences), with all proceeds raised via donation. Thus, the average cost per client was estimated at approximately $18,000. This amount covers salaries for therapists and support staff, therapy supplies, onsite security, meals, accommodations, and administrative costs. A breakdown of these costs (per person) is presented in Table 1. While over 50% of the budget is dedicated directly to therapy, the total program costs are used for this SROI calculation.

**Table 1:** Estimated average program costs (per person)

	Child Program	Adolescent Program
Staff salaries	$15,414.69	$12,686.82
Contract therapists	$958.80	$601.67
Therapy supplies	$167.37	$203.07
Insurance	$804.44	$487.15
Travel costs	$78.43	$18.13
Meals/snacks	$360.07	$221.52
Utilities and telephone	$654.93	$396.61
Repairs and maintenance	$256.59	$155.39
Onsite security	$1598.24	$967.87
**Total**	**$20,213.56**	**$15,738.23**

The BBR treats approximately 100 individuals annually (~ 60% under age 12). Thus, there is on average a 60:40 split of children to adolescents, which reflects both service demands and staffing; this ratio is used throughout the paper when discussing the proportion of individuals for whom any specific effect might apply.

### Theory of change

Our ultimate goal is to provide a treatment program that will improve the mental health and well-being of program participants and their families. This goal, however, rests on a few key assumptions: that participants will find value and meaning in the skills they learn; that providing a holistic, multimodal approach will provide the best chance for recovery; that parents and caregivers will accept the effects of treatment (which may be complex, and include some challenges); that participants will ultimately benefit from their involvement in the program (compared with other possible activities).

Our analysis of how well we are able to fulfill this goal is based on its impacts on key stakeholders in the system. Thus, for the purpose of this SROI, we identified the main stakeholders as:

- Children and adolescents – the direct recipients of the program, who need immediate assistance and may require help transitioning to adult responsibilities
- Family members – changes in the children receiving therapy will impact the home environment, while a requirement of participating in the program is broader family support
- School system – changes in the behaviour of the children ultimately affect the school community and their ability to function within this social network
- Criminal justice system – may need to become involved if problem behaviours escalate to the level of criminality
- Health and crisis prevention systems – may need to deal with both acute (critical incident follow up) and chronic health conditions associated with poor mental health

**Fig. 1:**
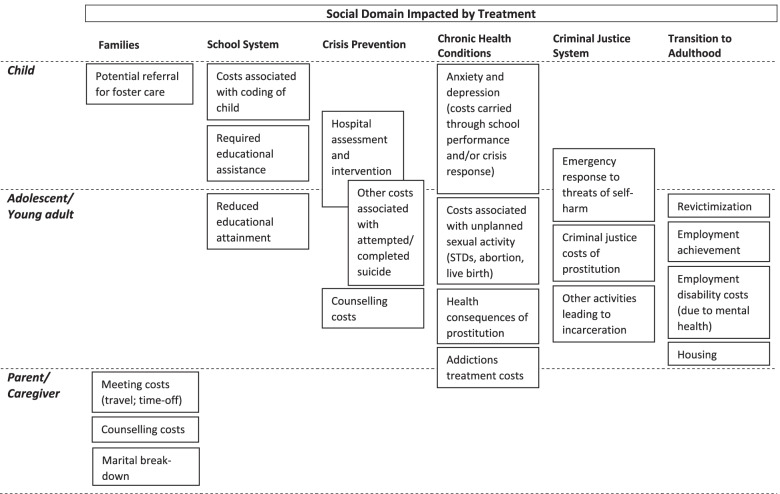
Impact inventory displaying areas of potential cost savings broken down by social domain. ^1^Health System impacts are limited to chronic conditions, as acute costs are presumed to be covered under Crisis Prevention Services, which may not be limited to health (e.g., emergency response by police services)

A societal perspective was taken in performing the analysis, inclusive of the stakeholders described above. To better understand the overall distribution of where the cost savings might be found and to capture which specific systems housed our stakeholder groups, an impact inventory was constructed to help evaluate the findings. Development of an impact inventory was one of the recommendations put forth for the reporting of cost-effectiveness analysis by the White Rose university consortium, to ensure that all potential consequences of an intervention are considered “regularly and comprehensively” [24]. This information is presented in Fig. 1.

### Monetizing outcomes

The SROI process first requires finding a proxy value for each presumed outcome under investigation. For example, one might assign a dollar value to “mental health” by calculating savings of fewer therapy visits per year. Outcomes were initially identified via a review of the literature and decided via team consensus. They were further confirmed via stakeholder discussions (described elsewhere) in which parents and caregivers communicated their experiences with the program. Approximately half our proxy values were obtained from the SROI Canada Proxy List, as assembled by the SiMPACT Strategy Group [25]; another quarter were obtained from the online Edmonton Financial Proxies list [26], and the rest in published research. Where necessary, present-day values were obtained using the Bank of Canada inflation calculator [27]. A complete list of indicators and their associated financial proxies is presented in Table 1.

These values were then multiplied by a weighting representing the proportion of individuals potentially affected. In our case, calculations applied either to children (60% of our population), adolescents (40%), or families as a whole (100%); see Table 2. For example, improvements in the ability to find a job post-graduation were limited to adolescents. Thus, proportion values specifically refer to the percentage of individuals within the program to whom the effect would apply.

**Table 2:** Financial proxies for identified domains

Identified Individual/Organization	Indicator	Duration (Years)	Financial Proxy	Proportion (%) Persons Affected	Proxy Value (Annual Per Person Cost)
**Adolescent/Young adult**	Individual at risk of suicide needing to attend fewer individual therapy sessions	1–5	Counselling session	16.6	$2292
	Reduced likelihood of short-term disability (improved productivity) required for mental illness due to stress and negative life circumstances	2–5	Cost of average short-term disability leave	8.0	$6805
	Reduced likelihood of long-term disability due to severe mental health consequences of PTSD and other chronic conditions	2–5	Cost of average long-term disability leave	4.0	$9970
	Reduced likelihood of turning to drugs and alcohol as a result of mental health symptoms associated with PTSD, anxiety, depression	2–5	Cost of addiction per addicted person (health, justice, social services)	17.0	$76,085
	Reduced risk of future sexual assaults, which the CDC has estimated at 33% for previous victims of rape	1–5	Cost of pain and suffering from (another) sexual assault	5.4	$100,463
	Increased probability of high school graduation and additional education	2–5	Cost of dropping out of high school (public, private, and intangible, per high school dropout per year)	16.0	$29,122
	Increased probability of finding and maintaining a higher paying job due to enhanced educational achievement	2–5	Annual salary difference between high school grad and individual with advanced degree or ticket	20.0	$17,068
	Decreased likelihood of requiring financial assistance with rent due to improved educational and work achievement	2–5	Monthly cost of Capital Housing Region - direct rent supplement	4.0	$6600
**Family**	Reduction in lost income due to time spent in meetings discussing issues	1	Alberta Minimum Wage^a^	100.0^b^	$510
	Reduction in time spent on phone by family discussing issues	1	Alberta Minimum Wage^a^	100.0^b^	$180
	Reduction in travel cost to families seeking mental health services within their geographic locale	1	Travel stipend/mileage for parents/guardians that qualify (12 monthly trips of 20 km/trip)	100.0^b^	$132
	Reduced probability of family pressure leading to alcoholism	1–5	Treatment for use of alcohol, based upon national average incl. Primary diagnosis and treatment	15.0	$7571
	Reduced probability of family pressure leading to divorce	1–5	Court costs for uncontested divorce (including filing and divorce papers)	2.8	$1353
	Reduced probability of family pressure leading to divorce	1–5	Court costs for contested divorce (including legal representation, trial costs, etc.)	0.7	$12,875
	Reduced probability of parents under pressure seeking parenting skills course to help deal with negative child behaviours	1–5	Cost of parenting skills course	25.0	$278
	Reduced probability of requiring family counselling to help deal with negative child behaviours and family dynamics	1–5	Cost of family counselling session	10.0	$1214
	Reduction in time of social worker to support the family’s ability to manage the target behavior at home	1–5	Salary of Social Worker	35.0	$5706
	Reduced probability of child needing to be placed into foster care	1–5	Caregiver rate schedule to cover all basic maintenance costs (per day)	10.0	$10,311
**School system**	Reduction in total teacher time spent attending to target behavior	1–5	Salary of Elementary School Teacher	6.0	$5974
	Funding for Educational Assistant, no longer req’d due to decreased behavioral issues	1–5	Average salary for an Educational Assistant	9.0	$13,748
	Funding for severe behavioral disability	1–5	Severe disability funding	9.0	$12,596
**Local authorities**	Reduced need for police assistance in crisis situations (as youth)	1–5	Police call out fee (Calgary rates)	13.9	$395
	Reduced need for police assistance in crisis situations (as youth)	1–5	Wage for police officers time taking care of suicide call (e.g., at ER)	13.9	$359
**Health system**	Reduced frequency of overdose and treatment for use of psychoactive substances	1–5	Cost of acute care medical stay for psychoactive drug use	7.5	$6017
	Reduced need for psychiatric risk assessment evaluation (as youth)	1–5	Cost of emergency room assessment	7.5	$266
	Reduced likelihood of psychiatric admission to hospital (as youth)	1–5	Cost of acute care medical stay for psychiatric condition	7.5	$10,555
	Reduced likelihood of suicide completion	1–5	Total direct and indirect costs associated with suicide completion	2.5	$1035
	Reduced likelihood of abortion	1–5	Cost of surgical abortion (equivalent of no insurance fees), 14–20 weeks	0.4	$3383
	Reduced likelihood of having an unplanned child	1–5	Average cost for uncomplicated delivery	0.3	$156
	Reduced likelihood of STDs due to sexual assault and/or risky relationships	1–5	Cost of STD testing and supplies, including staff costs (CDN dollars)	16.0	$36,595
	Reduced likelihood of turning to prostitution	1–5	HIV/AIDS - Direct costs of treatment, annually (British Columbia Centre for Excellence in HIV/AIDS estimates 1/4 of prostitutes in Vancouver are HIV+)	0.2	$15,915
**Criminal justice system**	Reduced likelihood of turning to prostitution	1–5	All direct and indirect social and human costs, but excluding tax evation (circulation of prostitution monies), reported elsewhere	0.7	$36,595
	Reduced likelihood of criminal justice involvement given stronger coping skills, heightened self-awareness, better self-regulation, etc.	1–5	Costs associated with 1 hr. youth court attendance, incl. Prosecution, legal aid, judge, court clerk, court security, and facility costs for the Provincial Court of Alberta	4.0	$8000
	Reduced likelihood of incarceration (as youth)	1–5	Costs associated with incarceration including administration, custody, parole services in Alberta (per day)	0.6	$15,111
	Reduced likelihood of turning to prostitution	1–5	Limited to costs directly affecting criminal justice via money laundering, tax evation (circulation of prostitution monies)	0.7	$38,983

^a^Note, this is likely a conservative estimate

^b^All families in treatment are considered treatment seeking, by definition; treatment course lasting one year

Next, probability values were applied, to reflect the relative risk of an individual experiencing said event. To consider a concrete example of this, while it would be predicted that some of the adolescents getting a job might need to receive short term disability benefits, this would only be a limited number of individuals – not the entire group. Clinical insight from a core team (including the Clinical Director of the BBR and the Director of the Scientific Advisory Committee) regarding previous rates were used to estimate probability values for commonplace events (e.g., the proportion of individuals attending therapy sessions). These were ultimately decided via team consensus, and informed by the battery of mental health questionnaires completed by applicants their parents upon entry to the program (described in Reeson et al., 2019 [15]). The general approach to this process was to generate a range of probabilities for any given event, debating the merits of the argument for or against a given estimate, and decreasing the value until all the group members agreed the new value was defensible. In this way, we tried to ensure that our estimates were inherently conservative (i.e., not guided by any extreme opinion). However, some events could be classed as rare-but-costly, but also highly noteworthy – making them prey to the availability heuristic (e.g., teen prostitution; teen pregnancy; completed suicide). To ensure we did not overestimate their occurrence, these were calculated using a combination of overall prevalence data, taking into account predicted rate elevations due to the children’s trauma histories (assessed via clinician interviews during intake), to determine the approximate probability of yearly incidence, as per the following equation [28].

Relationship between incidence and prevalence:

1 $$\mathrm P/\left(1-\mathrm P\right)=\mathrm{IR}\hspace{0.25em}\mathrm x\hspace{0.25em}\mathrm{Avg}.\;\hspace{0.25em}\text{Duration}$$

where P = affected proportion of the population and (1-P) is the proportion without it, IR = incidence rate, and Avg. Duration = average time that people have the condition (from diagnosis to cure/death).

These figures were then multiplied by the presumed duration (in years) during which the outcome in question is in effect. In this study, we utilized a 5-year time window, for the following reasons. First, most SROIs use a 5-year period, enabling more direct comparisons between studies [29–31]. Second, this window allows us to average out effects which may be greater during the first year (e.g., direct effects of therapy on family disruption) but may dissipate towards year five, presenting a more realistic estimate for an annual cost savings. Finally, it represents a conservative estimate, as some therapeutic effects potentially last decades (e.g., new skills that grow and reinforce themselves over time as one begins to adopt them as habits). Exceptions for the 5-year duration were made for effects that were specifically limited to the course of the treatment (which lasts one year), such as site visits, or those deemed unlikely to happen during the year the child was receiving therapy (e.g., incarceration); see Table 1.

Resultant values were then systematically reduced by several factors, which help account for potential devaluation. For example, certain changes experienced by the stakeholders might have “happened anyway”, while others naturally decrease over time. These are detailed below.

Attribution effects (effects that might be otherwise addressed by other providers) were systematically applied, with specialized care assumed to have more weight than generalized services. We assumed these effects would be limited to 20% maximum, since even mental health may not routinely provide services specific to a history of CSA. For instance, intake procedures may not adequately screen for adverse childhood events (ACEs) like CSA, and so may inadvertently put the child in situations which could be perceived as threatening given their history. They might ask the child to reveal information unknown to other family members that puts them at greater risk or ask sensitive questions in front of those involved in the abuse. Alternately, they may provide therapeutic modalities not adequately sensitive to the needs of a CSA victim. In all these cases, retraumatization is possible. For providers less versed in mental health, these missteps would be expected to be more severe, and they would likely ask even less information regarding the individual’s ACE history. Hence, for the different groups of service providers, attribution effects were modeled as follows:

**Table:** 

Family and social services	20%
Addictions and mental health	20%
Other health system providers	15%
Education	10%
Criminal justice	10%
Employment	10%
Housing	10%

A one-way sensitivity analysis was performed to test these assumptions. Specifically, projected figures for each category were both halved and doubled to examine the effects if other providers were able to meet the needs of our population more or less effectively than predicted. The SROI ratio was recalculated with these changes to determine both a conservative and a comprehensive estimate.

Deadweight, the amount of change that we believe “would have occurred anyway” (in the absence of the program) as a result of larger societal trends, was calculated with rates between 5 and 20%. Policing and criminal justice were given higher valuations (20%) to reflect the current “tough on crime” zeitgeist, while educational and health interventions were given lower values (5%) due to current lower levels of political support. Sexual abuse, prostitution and unwanted pregnancy were also given this valuation, due to their societal stigma. Mental health and addictions were given slightly higher values (10%) reflecting increased media exposure in recent years. All others were given 8% as an intermediate value.

We assumed treatment would not create negative effects elsewhere; thus, displacement was set at 0%, with a few specific exceptions. In terms of family problems, displacement was set at 20%, as it was hypothesized that improved patient functioning could, theoretically, lead to higher rates of family dysfunction. In these cases, improvement in the family member receiving treatment affects family dynamics in destabilizing ways, due to the other member’s own unresolved conflicts. This can cause an individual making therapeutic progress to be sabotaged in an effort to maintain a maladaptive, but predictable, home life [32]. Our 20% estimate predicts this kind of effect might be seen in 1 out of every 5 families. Additionally, recognizing that BBR is at a remote site, which creates complexities both in terms of travel and potential time from work to attend meetings which would likely not be there for regular outpatient visits, a 10% displacement cost was applied in these areas. Finally, we set displacement to 50% for adolescents. While not reflecting displacement per se, we wanted to capture the possibility that effective treatment could lead to higher use of mental health services; we predict that 1 in every 2 of the individuals in our program would want to continue therapy elsewhere, counterintuitively leading to increased costs to the healthcare system.

A 10% annual drop-off rate was assumed, to maintain consistency across measures. This figure was deemed reasonably conservative, as there may in fact be an increase in some therapeutic gains over time (e.g., with increased maturity, and a growing appreciation for new skills).

Finally, the value of change in future years was adjusted for estimated rates of future inflation (3% per annum), and a discount rate (5%) was applied to reflect future values in today’s terms. Finally, the total benefit over the five-year period was divided by the program cost to yield the SROI ratio.

## Results

The results of the SROI analysis for suggests that each dollar spent in CSA treatment results in an average cost savings of $11.60, over 5 years. Subtracting BBR treatment costs from total savings, the program produced a total average cost savings (net present value) per child of $192,197. The one-way sensitivity analysis, which examined plausible changes such as increasing (doubling) or decreasing (halving) each category of service provision replacing our services suggested that the SROI ratio would fall between 9.20–12.80.

We also wanted to better understand in which areas the majority of these savings were derived. In developing the impact inventory, we identified 6 key domains likely to experience cost savings via the program:

- Families: increased productivity and reduced time lost dealing with behavioural issues and treatment seeking; lower costs associated with discord in the household
- School system: reduced assessment and diagnosis costs, and associated behavioural management; improved academics as well as opportunity costs of not completing school
- Chronic health conditions: reduced risk of long-term health effects related to childhood trauma, including addictions and mental health; better life choices with long-term health implications (e.g., STDs associated with high-risk sexual behaviour)
- Crisis prevention: health savings from decreased risk of self-harm/suicide; lower associated social services costs and emergency services savings from police and ambulance
- Criminal justice system: decreased risk of deviant coping behaviours, such as drug use and prostitution, resulting in fewer cases before the courts; lower chances of incarceration
- Successful transition to adulthood: reduced costs associated with societal integration, such as finding and maintaining a stable home, family, and employment; decreased risk of another sexual assault/pregnancy, due to improved risk awareness, better coping skills, and self-care

**Fig. 2:**
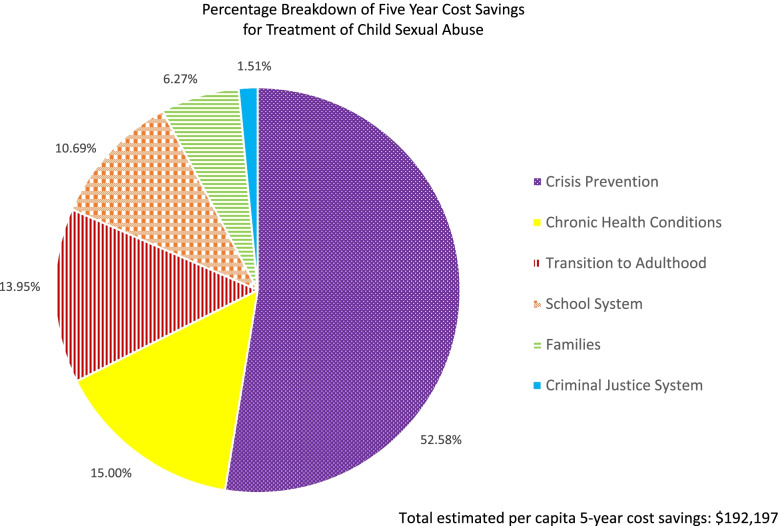
Total cost savings attributed to the treatment of CSA, as broken down by social domain

An analysis of the overall SROI ratio related to these groupings suggested that the domain of crisis prevention, related to decreased risk of self-harm and suicide prevention, produced the majority (53%) of the costs savings for this population. Costs for chronic health conditions and transitioning to adulthood each contributed roughly 15% of the cost savings. Together, impacts on the family and school system accounted for another 15%, while criminal justice only contributed ~ 1.5%. The relative cost savings attributed to the 6 social domains are presented in Fig. 2.

## Discussion

The BBR provides wraparound, therapeutic services to approximately 100 young survivors of CSA annually, housed onsite for 4 multi-week periods over the course of a year. Given the average cost for an individual receiving services at the BBR is estimated at $18,000, the average cost savings (total present value calculated) at the end of 5 years is $210,197 per child. The management of trauma due to CSA affected a number of domains and produced an SROI ratio of approximately 11.6:1. However, savings varied greatly between these domains.

Crisis prevention had the largest impact in terms of return on investment, with over 50% of the total value created. This mainly reflects the relatively disproportionate cost savings associated with averting even one suicide. Even in cases where suicide is not completed, higher costs associated with both mental health and addictions would be anticipated in the absence of treatment. The BBR program has, as one major focus, dealing with emotional dysregulation, with clients showing significant decreases in symptomatology after even the first round of treatment [15].

Another disproportionate impact is potential savings from a healthier lifestyle and improved coping skills, lessening the odds of sexual revictimization. This is a credible risk, as the National Center for Injury Prevention and Control has estimated that over 1/3 of women (35.2%) raped as a child experienced another rape in adulthood, double that of women who had not been raped (14%) [33]. Costs associated with even one such event are substantial; jury awards for pain and suffering associated with sexual assault exceed $100,000 in today’s dollars [34]. That therapy is likely to reduce this probability is supported by research suggesting that PTSD symptomatology per se appears to increase one’s risk of a subsequent attack [35, 36].

Similarly, prostitution carries not only costs for the criminal justice system (e.g. court costs and money laundering), but also health implications (increased risk of STDs, including HIV/AIDS), higher rates of unwanted pregnancy, and costs from increased risks of violence and/or addictions. Thus, while the odds of child from BBR entering prostitution are low (we estimated 0.65%), potential costs can be substantial.

However, these rare-but-costly events can and should not displace more routine costs associated with dealing with CSA on a daily basis, such as time spent by families in seeking out and consulting with specialists; struggles with educational/vocational achievement; and increased life stress. While the magnitude of these costs is much lower, they exact an ongoing toll on individuals and families. They may also be longer lasting and harder to mitigate. The BBR program employs a dyadic/familial focus for these reasons.

The fact that two areas of cost savings – transition to adulthood and chronic health conditions – both reflect longer-term impacts of CSA supports the notion that early treatment carries wide-ranging benefits for health and functioning. Meanwhile, influences on family and school functioning “right now” show relatively fewer effects. As per our earlier discussion about family functioning, this is not meant to imply that these areas are not of critical importance, simply that our means for measuring their impact (i.e., via proxy measures) are limited.

More surprising was the fact that effects on the criminal justice system were so minimal compared to the other social domains. This likely reflects the fact that, while there are potential negative effects in the criminal justice system related to a history of sexual abuse [37], most CSA victims do not turn to illegal methods to cope, or receive help via other systems (social supports and healthcare) in lieu of criminal justice, moderating direct impacts in this area.

There is reason to believe that many of the costs determined in this analysis are actually underestimates. For example, it is not unreasonable to suggest that health effects related to the ongoing stress of having an untreated child could result in health consequences for the parent or caregiver (e.g. chronic inflammation, hypertension, or cardiovascular problems). As another example, costs presented for foster care are related only to the per diem granted to a family to cover basic costs for caring for a child, however other allowable expenses (e.g., transportation, recreation, and additional supports) are also covered as required. In both of these situations, costs were limited to those that seemed the most moderate and defensible.

The SROI analysis provides a useful tool in exploring where social systems benefit from CSA treatment, however, it is difficult to link any specific therapeutic activity to a given effect. We attempted to calculate the overall effect of our services on both tangible measures and intangible social benefits. However, our recommendation would be not to focus on the final SROI ratio, but to use the information regarding domain allocations to inform both policy and practice.

## Limitations

Limitations of this study primarily reflect assumptions associated with the SROI process, such as the probability of an event occurring and hypothesized reactions to that event. As with most prevention studies, one of the main difficulties is the measurement of events that did not happen. Thus, for example, the evaluation of cost savings of a pregnancy that did not occur is predicated on everything from historical precedent, to local rates of teen pregnancy, to clinical observation, to the accuracy of expert opinions – in the end, the calculation of an exact figure relies upon the accuracy of these assumptions.

To this end, it must also be noted that this report was developed by members of the Research and Evaluation team at the BBR, which represent a potential conflict of interest and could call into question the objectivity of its conclusions. While every measure has been taken to ensure that our estimates were conservative and arrived at via consensus, it remains possible that our biases towards the inherent success of this program have coloured some of our predictions regarding probabilities of improvement related to program participation. This, in some sense, is a risk inherent to the SROI process, in which most often it is individuals conducting the SROI who are trying to catalogue potential value created by the program, and so have some measure of investment in the findings. Another issue is proxy attributions, which are inexact, may change over time, and are necessarily specific to the context in which the study is performed. There are numerous ways to measure cost savings, not all of which are available or obvious. As LeTourneau and colleagues observed [13], given such dissimilarities, including different methods in calculation, direct comparisons between SROI studies remain problematic. As such, our numbers may be generalizable only to Canada and similar environments.

It is also worth noting that some benefits and trade-offs likely interact with one another. For instance, one averted suicide attempt equals less time in hospital now and lower healthcare costs for psychological treatment later. But at the same time, other costs could shift upwards, because education and justice costs would actually decrease following a completed suicide. These so-called “knock-off effects” are nearly impossible to accurately predict, and so are effectively absent from our estimates, although the reader should be cognizant of them.

It should be kept in mind that the presumed impacts of this study were limited to a 5-year timeframe, presenting challenges in that it may both over- or under-represent some important effects. It is possible that most of the therapeutic benefits occur in the first year following therapy, and may be either forgotten or subsumed by other life habits after that. On the other hand, new coping and self-care skills may shift the lifetime trajectory with long-term benefits. 5 years was deemed to represent a fair compromise. However, this assumption does carry consequences for interpretation, since children in the lower age range would be just reaching adolescence by the end of that period, while the oldest would have reached early adulthood. The difference between the presumed life experiences of these groups is potentially vast. Moreover, it is also possible that medium- (3–5 year) and long-term (10–15 year) follow up could reveal a pattern of both improvements in some areas, but diminishing effects in others. Future research will attempt to explore this complex subject. Finally, it is difficult to compare different CSA treatment programs and approaches. The BBR utilizes an integrated model incorporating a variety of evidence-based treatment philosophies and approaches, with repeated treatment episodes over the course of a year. By comparison, other facilities treating CSA may focus only on individual or group therapy, or therapy plus medication [38]. While it is difficult to pinpoint how any given therapy relates to a specific outcome, this also means that treatment at BBR, while more expensive, is potentially more cost effective than less holistic therapies.

## Conclusion

In a healthcare space marked by competition for limited budgetary resources, it is crucial to determine the return on investment of services for CSA - a condition which is often hidden and neglected in terms of both funding and priority. It has been observed that “changing the conversation from cost to value (impact) will offer greater insight into the real value of healthcare programs and the differences they make” [22]. Accordingly, our results support the need for specialized, evidence-based services for survivors of CSA, and highlight the cost effectiveness of this approach. Our ability to intervene early in development, potentially alleviating the outcomes of severe, costly, negative events, provides a good prospect for savings in terms of both monetary cost and emotional toll. Given the relatively high SROI achieved (in excess of 11:1), it seems clear that although these services appear “expensive” the cost savings more than offset the investment. Moreover, funding reallocations may assist with their implementation, representing a strong opportunity for governments to achieve value-for-money. This is essential for problems as widespread and far-reaching as CSA. We submit finding an SROI ratio for the treatment of CSA represents an important step for advocacy efforts worldwide.

## Data Availability

The data that support the findings of this study are available from the sponsor (Little Warriors BBR) but restrictions apply to the availability of these data, which were used under license for the current study, and so are not publicly available. Data are however available from the authors upon reasonable request and with permission of the BBR. More information on the data used for analysis can be obtained by contacting the lead author on reasonable request.
